# Optical Fiber Bundle-Based High-Speed and Precise Micro-Scanning for Image High-Resolution Reconstruction

**DOI:** 10.3390/s22010127

**Published:** 2021-12-25

**Authors:** Jiali Jiang, Xin Zhou, Jiaying Liu, Likang Pan, Ziting Pan, Fan Zou, Ziqiang Li, Feng Li, Xiaoyu Ma, Chao Geng, Jing Zuo, Xinyang Li

**Affiliations:** 1 Key Laboratory on Adaptive Optics, Chinese Academy of Sciences, Chengdu 610209, China; jiangjiali@ioe.ac.cn (J.J.); zhouxin201@mails.ucas.ac.cn (X.Z.); liujiaying16@mails.ucas.ac.cn (J.L.); panlikang20@mails.ucas.ac.cn (L.P.); panziting20@mails.ucas.ac.cn (Z.P.); zoufan19@mails.ucas.ac.cn (F.Z.); liziqiang16@mails.ucas.edu.cn (Z.L.); lifeng@ioe.ac.cn (F.L.); zuojing17@mails.ucas.ac.cn (J.Z.); xyli@ioe.ac.cn (X.L.); 2 Institute of Optics and Electronics, Chinese Academy of Sciences, Chengdu 610209, China; 3 College of Materials Science and Opto-Electronic Technology, Chinese Academy of Sciences, Beijing 100049, China; 4 Chengdu Institute, Sichuan University of Arts and Science, Dazhou 635000, China; maxiaoyu@sasu.edu.cn

**Keywords:** optical fiber bundle, high-resolution, piezoelectric-ceramic-chip, micro-scanning

## Abstract

We propose an imaging method based on optical fiber bundle combined with micro-scanning technique for improving image quality without complex image reconstruction algorithms. In the proposed method, a piezoelectric-ceramic-chip is used as the micro-displacement driver of the optical fiber bundle, which has the advantages of small volume, fast response speed and high precision. The corresponding displacement of the optical fiber bundle can be generated by precise voltage controlling. An optical fiber bundle with core/cladding diameter 4/80 μm and hexagonal arrangement is used to scan the 1951 USAF target. The scanning step is 1 μm, which is equivalent to the diffraction limit resolution of the optical system. The corresponding information is recorded at high speed through photo-detectors and a high-resolution image is obtained by image stitching processing. The minimum distinguishable stripe width of the proposed imaging technique with piezoelectric-ceramic-chip driven micro-scanning is approximately 2.1 μm, which is 1 time higher than that of direct imaging with a CCD camera whose pixel size is close to the fiber core size. The experimental results indicate that the optical fiber bundle combined with piezoelectric-ceramic-chip driven micro-scanning is a high-speed and high-precision technique for high-resolution imaging.

## 1. Introduction

As a passive device that can be twisted and bent flexibly, the optical fiber image bundle has demonstrated its success in precision optical detection due to its advantages of light weight, resistance to electromagnetic interference and easy realization in complex spatial structures [1,2,3,4,5,6,7,8]. The optical fiber image bundle is composed of multiple fibers with cores and claddings. The fiber cores served as effective pixels that can transmit image information from a distance to the proximal end of the apparatus. In this case there exists a pixilation artifact, which is due to the use of individual fibers as image pixels [9]. The inherent limitations of their physical structure block information and degrade resolution, resulting in blurred imaging of target [10]. The limitations degrading image quality include cross talk between adjacent cores and the honeycomb pixelation caused by lack of information in cladding regions [11,12].

Therefore, to improve imaging quality of optical fiber bundle imaging technique, it is necessary to explore appropriate methods to suppress the impact of honeycomb patterns and compensate for the missing cladding information. Several approaches have been proposed to overcome these limitations. In-depth analyses over fiber bundles have been performed both theoretically and experimentally based on the fiber parameters (core size, core spacing, NA, refractive indices) [13], micro-scanning modes (rotating refractor, optical space-fed phased arrays) [14,15] and image reconstruction algorithms (deep learning, blind deconvolution, maximum a posteriori estimation) [10,16,17,18,19] to thoroughly characterize the properties of the fiber imaging [10,13,20,21]. Lee et al. have taken advantage of precise motion control to overlap four images obtained by lateral shift, recovering some hidden information and eliminating honeycomb patterns [22]. Shao et al. enhanced the resolution for optical fiber bundle imaging by using maximum a posteriori estimation [10]. Cheon et al. proposed a spatial compounding method and optimized an image registration process, which can register random transverse images [12]. In addition, micro-scanning technique utilizing sub-pixel displacement to obtain multiple images in the same scene, resulted in high-resolution images [23]. Sui et al. suggested the spatial resolution of images could be improved through a flat optical component based micro-scanning technique [14]. However, the determination of dip angle of the micro-scanning germanium lens based on the flat optical components was strict and difficult due to the influence of too many factors. Shi et al. developed a cubic convolution method and implemented reconstruction and restoration from micro-scanned images [24].

In this study, we combine optical fiber bundle with micro-scanning driven by piezoelectric-ceramic-chip to realize high resolution imaging, for example in narrow space, harsh environment, or environment with strong electromagnetic interference, etc. We first describe the principle of micro-scanning based optical fiber bundle imaging. Via scanning the optical fiber bundle can be shifted horizontally or vertically to collect information that is originally located in the cladding region. The discrete sampling modulation transfer function and the integral sampling modulation transfer function of the fibers are analyzed in the spatial-frequency domain, and it is used as the figure of merit for a quantitative investigation of the relationship between micro-scanned image quality and scanning step. We then analyze the influence of micro-scanning step on the image quality to determine the optimum micro-scanning mode. Compared to previous micro-scanning techniques [25,26], the sub-image scanned by each fiber is a high-resolution image, with its resolution limited only by the diffraction limit of the optical system, not the size of the fiber cores. In addition, a home-made piezoelectric-ceramic-chip, which has the advantages of small volume and fast response speed, is used as the micro-displacement driver [27,28]. The micro-scanning displacement of the imaging system can be realized by precise voltage control to the piezoelectric-ceramic-chip. Moreover, experiment is performed to compare the performance of optical fiber bundles based micro-scanning imaging and the direct CCD camera imaging of 1951 USAF target. The results demonstrate that the proposed imaging approach is promising in realizing high-speed and high-resolution imaging.

## 2. Theoretical Analysis

**Figure 1:**
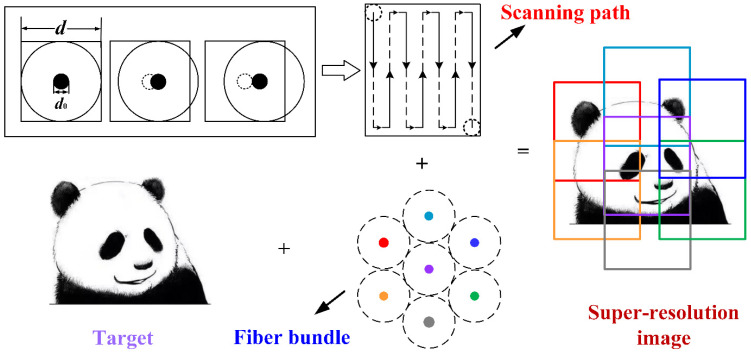
Schematic of high-resolution imaging based on optical fiber bundle combined with micro-scanning.

The schematic of the high-resolution imaging technique based on optical fiber bundle combined with micro-scanning is shown in Figure 1. The area where an optical fiber located is assumed to be the size of a pixel (*d*), and the fiber core (*d*_0_) is regarded as an effective pixel. The optical fiber bundle is densely arranged into a hexagon, and the image information is mainly obtained by the core. Driven by the piezoelectric-ceramic-chip, each optical fiber scans a corresponding area of the target continuously to obtain multiple sub-images. A large-field image of the target is obtained by stitching the sub-images.

In order to quantitatively investigate the relationship between the image quality and the micro-scanning mode, a modulation transfer function [*MTF*] is introduced as the evaluation metric. It is known that the optical fiber imaging bundle is a discrete sampling imaging device. It is regarded as a spatially invariant linear system because of local isoplanatic property of the single optical fiber. The fiber optic image transmission process includes integral sampling of the fiber core and discrete sampling of each fiber with dense arrangement. The modulation transfer function [*MTF*_fib_] is composed of the product of the fiber integral function [*MTF*_fib-int_] and the sampling function [*MTF*_fib-samp_]. Under the premise of constant optical system, the image quality of the system depends on the image transmission quality of the fiber bundle, which can be described as [*MTF*_fib_]:

(1) $$MTF_{\mathrm{fib}}=MTF_{\mathrm{fib-int}}\cdot MTF_{\mathrm{fib-samp}},$$

The integral function [*MTF*_fib-int_] of the optical fiber bundle is expressed by the Fourier transform of the circular function:

(2) $$MTF_{\mathrm{fib-int}}=ℱ\left[circ\left(\frac{\rho }{d_{0}/2}\right)\right]=\left|\frac{d_{0}J_{1}\left(\pi d_{0}f_{x}{}_{,y}\right)}{2f_{x}{}_{,y}}\right|,$$

The sampling function *MTF*_fib-samp_ is described by the Fourier transform of the rectangular function:

(3) $$MTF_{\mathrm{fib-samp}}=\left|ℱ\left[rect\left(\frac{x}{\Delta },\frac{y}{\Delta }\right)\right]\right|=\left|\frac{\sin\left(\pi \Delta \cdot f_{x}\right)}{\pi \Delta \cdot f_{x}}\cdot \frac{\sin\left(\pi \Delta \cdot f_{y}\right)}{\pi \Delta \cdot f_{y}}\right|\;\;=\left|\sin c\left(\Delta \cdot f_{x}\right)\cdot \sin c\left(\Delta \cdot f_{y}\right)\right|,$$

Among them, *d*_0_ is the diameter of the fiber core. $\rho =\sqrt{x^{2}+y^{2}}$ is the curvature radius. The $f_{x}$ and $f_{y}$ represent spatial frequency in the x and y directions, respectively. *J*_1_ is the first-order Bessel function of the first kind. Δ is the sampling step of the optical fiber beam.

Therefore, *MTF*_fib_ is expressed as:

(4) $$MTF_{\mathrm{fib}}=MTF_{\mathrm{fib-int}}\cdot MTF_{\mathrm{fib-samp}}\;\;=\left|\frac{d_{0}J_{1}\left(\pi d_{0}f\right)}{2f}\right|\cdot \left|\sin c\left(\Delta \cdot f_{x}\right)\cdot \sin c\left(\Delta \cdot f_{y}\right)\right|,$$

**Figure 2:**
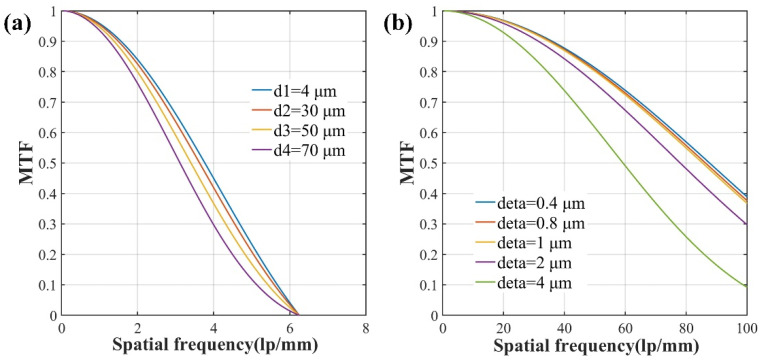
The dependences of modulation transfer function (*MTF*_fib_) on (**a**) core diameter and (**b**) scanning step.

The *MTF* of system [*MTF*_sys_] depends on *MTF*_fib_ under the premise of determinate optical system. *MTF*_fib_ is calculated with fiber cladding diameter of 80 μm and core diameter of 4 μm, 30 μm, 50 μm and 70 μm, respectively, and the results are presented in Figure 2a. It is depicted that under the same spatial frequency, a fiber bundle with a smaller core diameter takes on a larger *MTF*_fib_ and results in a higher image resolution.

It is well known that multimode optical fibers are mostly used in fiber bundle imaging to alleviate the pixelation effect and other defects [29,30]. However, when the core-cladding ratio is increased to a certain limit, thin cladding layers also result in larger optical crosstalk between adjacent fiber cores [31]. Therefore, taking into consideration the manufacturing process, scanning speed, pixel resolution and expansibility for single-mode fiber bundle, 4/80 μm core/cladding fiber (small core, thin cladding) is chosen to form the fiber bundle in our experiment. As shown in Figure 2b, *MTF* of the fiber bundle with core diameter of 4 μm and cladding diameter of 80 μm is calculated when the scanning step (Δ) is set to 0.4, 0.8, 1, 2 and 4 μm, respectively. It can be seen that the smaller the scanning step, the higher the corresponding spatial frequency, and the higher the resolution of the fiber bundle imaging. 

It is reported that to obtain a high-resolution image, the temporal resolution is sacrificed. However, Figure 2b shows that with the decreasing sampling step, the spatial frequency changes little and *MTF* curves almost coincide. From the results presented in Figure 2b, the scanning step should be chosen to compromise the temporal resolution and the spatial resolution.

## 3. Experimental Setup 

**Figure 3:**
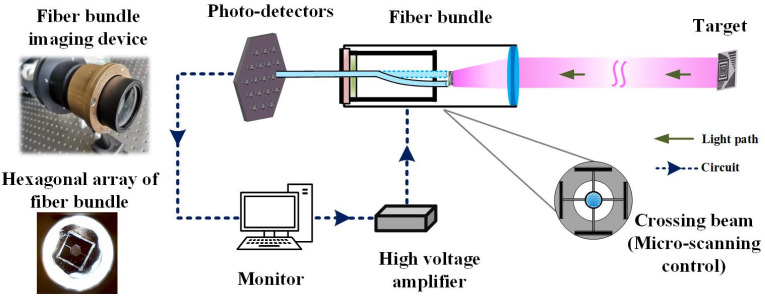
Experimental schematic of high-resolution image based on optical fiber bundles combined with micro-scanning.

The experimental setup is presented in Figure 3. The target is placed at infinity and imaged at like square focal plane of the lens. The fiber bundle is composed of seven optical fibers closely packed with a hexagonal structure. The core diameter of each fiber is 4 μm and the cladding diameter is 80 μm. It is inserted into a homemade micro-displacement driving device, which is composed of flexible crossing beam with piezoelectric-ceramic-chip. They are placed at the focal point of the camera lens to obtain the image of the target. The piezoelectric-ceramic-chip is actuated by a multi-channel high-voltage amplifier (Home-made) and the required deformation is precisely controlled by the output voltage signal. The optical fiber bundle is forced to shift in x and y directions, and a two-dimensional image is recorded. The scanning step can be adjusted to achieve less than or equal to the resolution limit of optical system by modulating the output voltage signal. The image information transmitted by the optical fiber bundle is captured by photo-detectors (PDs, Thorlabs, PDA36A-EC) at high speed and recorded by a computer for image processing.

**Figure 4:**
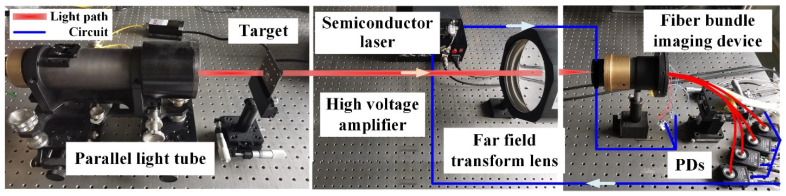
Photos of fiber bundle-based super-resolution imaging driving by micro-scanning.

As shown in Figure 4, the target used in the experiment is an American standard USAF 1951 (Daheng Optics, United States Air Force target) resolution board, which is suitable for evaluating the resolution of the lens and the distortion of the imaging system. It is placed at the focal point of transforming lens to assume to be at infinity. The aperture diameter and focal length of transforming lens and camera lens are *D*_1_ = 200 mm, *ƒ*_1_ = 1 m and *D*_2_ = 44 mm, *ƒ*_2_ = 55 mm, respectively. The beam emitted by the semiconductor laser (MIR-III-650 L-50 mW) with 650 nm wavelength illuminates the target through a parallel tube (Home-made). And the target is imaged on the input end of the optical fiber bundle. According to the Rayleigh criterion, the diffraction limit of the optical system is approximately 1 μm.

**Figure 5:**
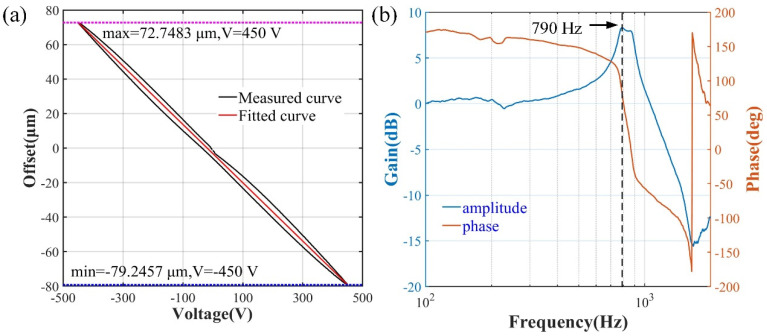
The performance of micro-displacement driving device: (**a**) offset range of the optical fiber bundle as the function of applied driving voltage; (**b**) frequency response curve.

The inductance micrometer (Zhongyuan Measuring, DGB-5B) is used to test the dynamic range of the optical fiber bundle driven by the piezoelectric-ceramic-chips in the x and y directions. The maximum offsets of fiber bundle are −79~73 μm (as plotted in Figure 5a) and −58~62 μm, when the applied driving voltages for both directions are in the range of ±450 V. The first resonance-frequency of the driving device is approximately 790 Hz, as depicted in Figure 5b.

## 4. Results and Discussion

### 4.1. Micro-Scanning Step Size

**Figure 6:**
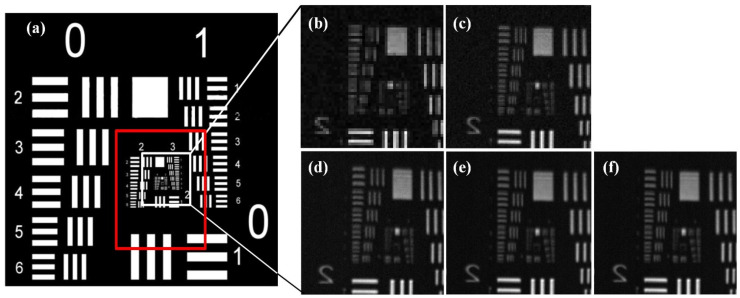
The target and sub-images obtained with different scanning steps. (**a**) USAF 1951 resolution board; scanning steps of (**b**) ~4 μm, (**c**) ~2 μm, (**d**) ~1 μm, (**e**) ~0.8 μm, and (**f**) ~0.4 μm, respectively.

Experiments are performed with different scanning steps of ~4, ~2, ~1, ~0.8 and ~0.4 μm. The sub-images obtained by the same optical fiber under different scanning step settings are recorded. Combined with the simulation results presented in Figure 2b, the experimental results show that when the core-diameter is 4 μm, the sub-image with scanning step ~4 μm, as presented in Figure 6b, is slightly blurred, while the sub-image shown in Figure 6c with ~2 μm scanning step presents a pixel block effect. On the other hand, the sub-images obtained with scanning steps ~1, ~0.8 and ~0.4 μm, as shown in Figure 6d–f, respectively, are clear, and their *MTF* curves are almost identical. Theoretically, when the system’s scanning step reaches the diffraction limit resolution, further decreasing the scanning step will not improve the image quality. Therefore, in the experiments the scanning step is set to 1 μm for collecting the target images for optimal temporal resolution and spatial resolution.

### 4.2. High-Resolution Image Acquisition and Stitching

**Figure 7:**
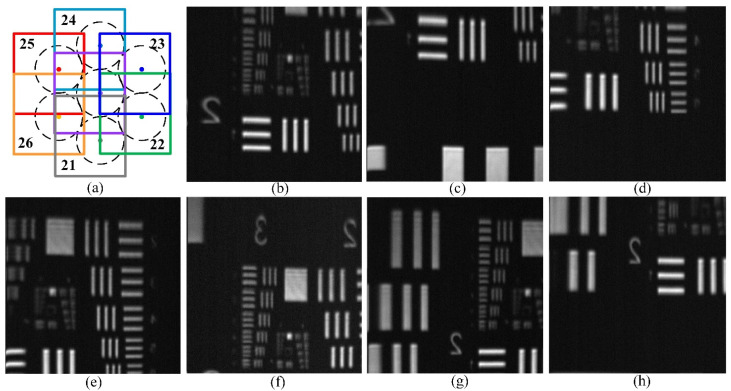
Scanning sub-images of each fiber with 160 × 160 points and scanning step of ~1 μm. (**a**) Scanning optical fiber arrangement; (**b**) purple-middle; (**c**) gray-21; (**d**) green-22; (**e**) blue-23; (**f**) cyan-24; (**g**) red-25; (**h**) orange-26.

Seven adjacent fibers of the optical fiber bundle are used to scan the center pattern of the USAF 1951 resolution board. The arrangement of the seven fibers is shown in Figure 7a, and the scanning path is vertical. The applied driving voltage is from −450 V to 450 V with scanning step of 1 μm, which results in scanning points of about 160 × 160. Figure 7 presents the corresponding sub-images of each fiber.

**Figure 8:**
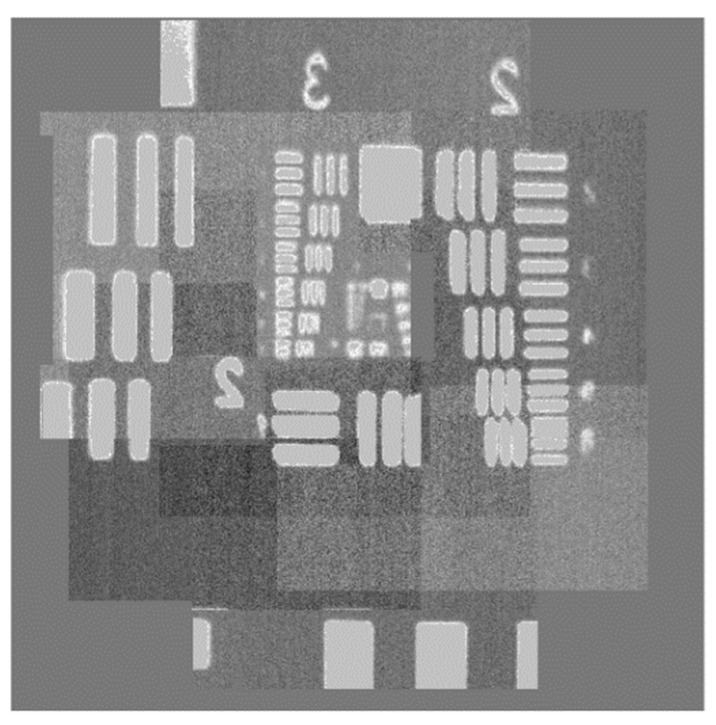
Stitched high-resolution image.

Taking into consideration the hexagon structure of the fiber bundle, the vertical spacing between adjacent fibers actually is *d*_y_ = 80 μm, and the horizontal spacing is *d*_x_ = 70 μm. However, the maximum scanning ranges become −58~62 μm and −79~73 μm in both directions, respectively, as presented in Figure 5. It can be seen that the sub-images obtained by adjacent fibers within this driving voltage range overlap. Hence, the sub-images need to be segmented in order to receive better stitching effect. Based on the displacement of optical fiber bundles under the driving voltage and the actual offset required to compensate for the cladding information, we calculate the size of non-overlapping sub-images and construct a sufficiently large matrix sequence to place the sub-images obtained by each fiber in the corresponding position. The final stitched image obtained by the system is shown in Figure 8.

### 4.3. Image Quality Evaluation 

Comparative experiments are performed to investigate the capability of high-resolution imaging of the proposed imaging technique. The experimental parameters are presented in Table 1. A high-resolution CCD camera (Spiricon, SP620U) with a pixel pitch of 4.4 μm × 4.4 μm, with the pixel size close to the core size of the fiber bundle, is selected to obtain regular image by placing the CCD camera at the focal plane of the system. 

**Figure 9:**
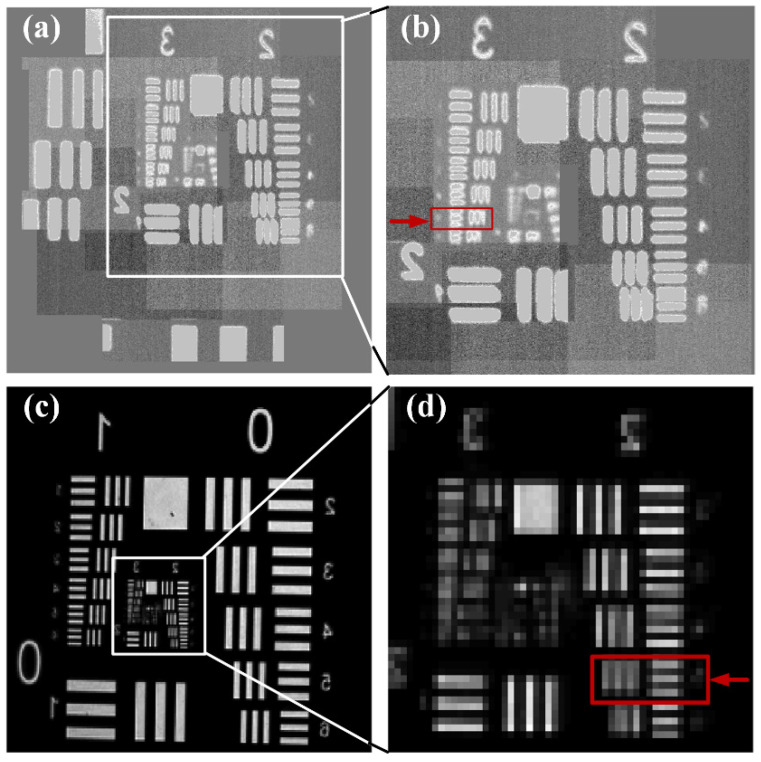
Comparison of stitching imaging and direct imaging with a CCD camera. (**a**) Original stitching image obtained by the proposed imaging technique; (**b**) enlarged middle view of the stitching image; (**c**) direct image obtained by the CCD camera; (**d**) enlarged CCD image of the same area as (**b**).

The USAF 1951 resolution board is directly imaged by the camera, and the resulted image is presented in Figure 9c. The stitching image obtained by the proposed imaging technique is presented in Figure 9a. In order to intuitively compare the imaging performance of both techniques, a same area is intercepted for comparison, as shown in Figure 9b,d. It is clearly found that the image quality captured by the CCD camera is somewhat blurred as compared to that obtained by the proposed method.

From Appendix A, the minimum fringe that can be resolved by the proposed imaging system if optical fiber bundles combining with micro-scanning is the fifth row of the third group, which is 12.7 lp/mm, and the corresponding line width is 39.37 μm. From the object-image relationship, the distinguishable fringe length of the proposed imaging system is approximately 2.1 μm. The fringe contrast here is 0.283. The smallest stripe that can be distinguished by direct imaging with CCD camera is the fifth stripe pair of the second group, which is 6.35 lp/mm, corresponding to a line width of 78.75 μm, and the resolution is approximately 4.4 μm by calculation. And the corresponding fringe contrast is 0.186. The image resolution achieved by combination of the optical fiber bundle and micro-scanning is improved by at least 1× compared with the direct image obtained by the CCD camera. At the same time, under this premise, the fringe contrast of the scanned image is 1.5× that of the image obtained by the CCD. The result proves the feasibility of high speed, high precision and high-resolution image reconstruction by the optical fiber bundle combined with micro-scanning technique. However, the edge of stripes is somewhat broadened due to scanning angle and image stitching. It could be restored by finding suitable displacement range and optimal image processing algorithm to get high quality images with sharper edge in the future.

## 5. Conclusions

In summary, we have demonstrated experimentally the feasibility of high-resolution imaging via the optical fiber bundles with micro-scanning. The resolution of the scanned images was promoted with the decrease of scanning step on the premise of a certain size of fiber core and cladding. The multiple sub-images were acquired simultaneously through one scan, and the number of sub-images were determined by the number of fibers in the fiber bundle. It meant that high-speed, wide-field imaging should be achievable by increasing the number of fibers. In addition, the sub-image scanned by each fiber is a high-resolution image. The USAF 1951 resolution board was precisely scanned using piezoelectric-ceramic-chips and the resulting sub-images were recorded through photo-detectors at high speed. A combined high-resolution image was acquired by splicing the recorded sub-images. The result indicated that the minimum distinguishable fringe was approximately 2.1 μm when the scanning step was 1 μm. Compared with the direct imaging by CCD camera with similar pixel size (4.4 μm × 4.4 μm), the resolution was improved by approximately 1 time. Further research will be focused on the image registration algorithm optimization and large-field and high-resolution imaging applications of the proposed imaging technique based on optical fiber bundles with micro-scanning.

## Figures and Tables

**Table 1:** Parameters of micro-scanning and direct imaging in the experiment.

	Parameters	Value
**Optical fiber lenses**	Focus length	55 mm
	Aperture diameter	44 mm
**Micro-scanning system**	Driving voltage	−450 V–450 V
	Scanning step size	~1 μm
	Scanning steps	160 × 160
	Scanning range	x: −58 μm~62 μm
		y: −79 μm~73 μm
**Optical fiber bundles**	Core/cladding	4 μm/80 μm
	Wavelength	650 nm
	Arrangement	Hexagon
	Number of fibers	7
**CCD**	Pixel size	4.4 μm × 4.4 μm

## Data Availability

The parameters of USAF 1951 resolution board used in Section 4.3 were listed in Table A1.

## Appendix A

The parameters of USAF 1951 resolution board used in Section 4.3 were listed in Table A1.

**Table A1:** Identification plate/resolution test panel (USAF 1951).

Group Number	Line Pair (Lp) Line Width (Lw)/μm	Series Number					
		1	2	3	4	5	6
**0**	Lp/mm	1	1.12	1.26	1.41	1.59	1.78
	Lw	500	445.45	396.85	353.55	314.98	280.62
**1**	Lp/mm	2	2.24	2.52	2.83	3.17	3.56
	Lw	250	222.72	198.43	176.78	157.49	140.31
**2**	Lp/mm	4	4.49	5.04	5.66	6.35	7.13
	Lw	125	111.36	99.21	88.39	78.75	70.15
**3**	Lp/mm	8	8.98	10.1	11.3	12.7	14.3
	Lw	62.5	55.68	49.61	44.19	39.37	35.08
**4**	Lp/mm	16	17.95	20.16	22.62	25.39	28.5
	Lw	31.25	27.84	24.8	22.10	19.69	17.54
**5**	Lp/mm	32	36	40.3	45.3	50.8	57
	Lw	15.63	13.92	12.4	11.05	9.84	8.77
